# Monitoring and modeling non-native invasive green iguana population response to harvesting on Grand Cayman, Cayman Islands

**DOI:** 10.1007/s10530-022-02828-0

**Published:** 2022-06-04

**Authors:** Frank F. Rivera-Milán, Jane Haakonsson, Vaughn Bodden, TayVanis Oyog, Sophie O’Hehir

**Affiliations:** 1 Branch of Assessment and Decision Support, Division of Migratory Bird Management, United States Fish and Wildlife Service, Laurel, MD 20708 USA; 2 Department of Environment, Cayman Islands Government, Grand Cayman, KY 1002 Cayman Islands

**Keywords:** Abundance, Estimation, Harvest, *Iguana iguana*, Prediction

## Abstract

Registered hunters harvested over 1.3 million non-native invasive green iguanas (*Iguana iguana*) on Grand Cayman between October 2018 and August 2021. We used islandwide post-reproduction survey-based abundance estimates in August 2014–2021 and model-based abundance predictions for August 2022–2030 to assist natural resource managers with reassessment and modification of the harvest strategy due to diminishing returns to hunters paid per green iguana harvested. We need harvest rates > 0.600 for desired abundance ≤ 50,000 and > 0.700 for desired abundance ≤ 10,000 green iguanas. With harvest rates < 0.600, the population would likely remain above desired abundance. Without harvesting, the green iguana population would likely reach carrying capacity by August 2026.

## Introduction

The non-native invasive green iguana (*Iguana iguana*) was first noticed on Grand Cayman in the early 1990s (F. Burton, personal observation). Estimated abundance surpassed 1.3 million in August 2018 (see Rivera-Milán and Haakonsson 2020: Table 2). Concerned about the negative impacts of overabundance, natural resource managers from the Cayman Islands Government contracted the services of a private company and registered hunters to harvest green iguanas. Registered hunters harvested over 1.3 million green iguanas on Grand Cayman between October 2018 and August 2021. However, the total number of green iguanas harvested per month decreased from 154,829 in November 2018 to 7200 in August 2021 (https://doe.ky/green-iguana-cull-updates/). To boost hunting activity, natural resource managers wanted to reassess and modify the harvest strategy from a bounty system where hunters are paid per green iguana harvested to a system in which contracted hunter teams are paid for the total number of hunting hours per week (F. Burton, personal communication). In addition, based on islandwide post-reproduction surveys in August 2020 and 2021, they wanted to lower desired abundance from ≤ 50,000 to ≤ 10,000 green iguanas, and they wanted to simulate the population trajectory without harvesting and with variable harvest rates. Here, we provide that information using abundance estimates from islandwide post-reproduction surveys conducted annually in August 2014–2021. Our objectives were to (1) fit a Bayesian state-space logistic model with August 2014–2021 survey-based abundance estimates, (2) update posterior estimates of population carrying capacity (*K*), maximum intrinsic rate of population growth (*r*_*max*_) and maximum sustained harvest rate (*h*_*msy*_ = *r*_*max*_/2), and (3) predict future post-reproduction abundance in August without harvesting and with harvest rates between 0.001 and 0.900 during 2022–2030.

## Methods

We counted hatchlings, juveniles and adults at 157–212 fixed point locations along and away from roads surveyed annually on Grand Cayman in August 2014–2021 (see Rivera-Milán and Haakonsson 2020: Fig. 1). We used distance sampling and *N*-mixture models to estimate abundance (Buckland et al. 2015; Kéry and Royle 2016). We fit a Bayesian state-space logistic model with abundance estimates, corrected for changes in detection probability due to observer detectability and green iguana availability (see Rivera-Milán and Haakonsson 2020: Table 1). The Bayesian state-space logistic model accounted for observation error (e.g., due to imperfect detection and partial control over harvesting) and process variance (e.g., due to environmental stochasticity and incomplete understanding of population dynamics).

**Fig. 1:**
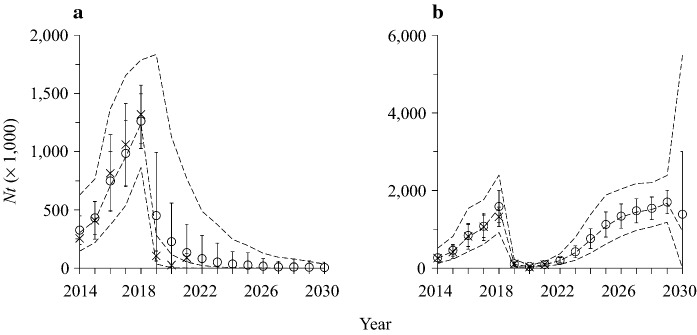
Grand Cayman green iguana survey-based abundance estimates (crosses with vertical lines for means and standard errors) and model-based posterior estimates of predicted abundance (circles with vertical lines for means and standard deviations and dashed lines for medians and 2.5–97.5th percentiles) with **a** harvest rate *h* ~ Uniform (0.701, 0.900) and **b** without harvesting during 2022–2030

**Table 1:** Green iguana survey-based abundance estimates on Grand Cayman in August 2014–2021, based on distance sampling analysis

Date	Mean^a^	SE^b^	95% CI^c^	
08/2014	254,162	105,725	116,160	556,116
08/2015	408,749	161,343	193,887	861,719
08/ 2016	814,855	331,218	378,635	1,753,838
08/2017	1,060,687	353,234	562,735	2,002,824
08/2018	1,319,939	252,108	910,806	1,912,854
08/2019	103,020	42,925	47,027	225,683
08/2020	25,259	9,485	12,395	51,473
08/2021	87,751	34,706	41,568	185,250

^a^Estimated abundance in 19,600 hectares (age and sex classes combined)

^b^Bootstrapped standard errors

^c^Lognormal 95% confidence intervals

In the model (see Rivera-Milán and Haakonsson 2020: Eq. 2), parameter *K* represents the abundance above which the green iguana population tends to decline due to density dependence (e.g., competition for limited space to reproduce). Parameter *r*_*max*_ is the exponential rate of increase of the population at low density and under favorable conditions (e.g., with plenty of space and other resources needed to maximize reproductive output). Parameter *N*_*t*_ is the true unknown abundance state of the population and *H*_*t*_ is the total number of green iguanas harvested in time period *t*. That is, total harvest (*H*_*t*_) = *N*_*t*_*h*_*t*_, where *h*_*t*_ is the harvest rate generated as part of the Markov chain Monte Carlo algorithm using uniform prior distributions for six hypothetical harvesting scenarios: *h* ~ Uniform (0.001, 0.100), (0.101, 0.300), (0.301, 0.500), (0.501, 0.700), (0.701, 0.900), and (0.001, 0.900). Based on previous modeling simulations presented in Rivera-Milán and Haakonsson (2020, Table 3), we also used uniform priors to update posterior estimates of parameters *K* ~ Uniform (900,000, 2,000,000) and *r*_*max*_ ~ Uniform (0.500, 2.000). For additional information about population monitoring and modeling methods, see Rivera-Milán and Haakonsson (2020).

## Results and discussion

**Fig. 2:**
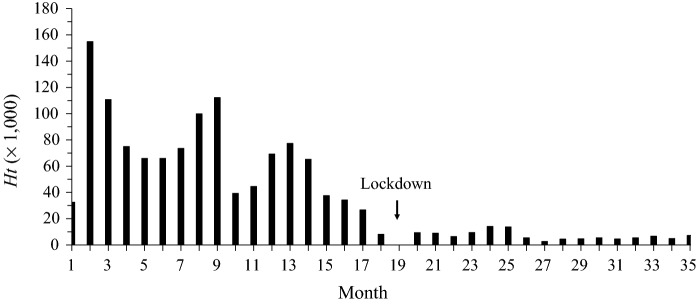
Total number of green iguanas harvested per month on Grand Cayman between October 2018 and August 2021

In Table 1 and Fig. 1a, b, we provide survey-based abundance estimates for August 2014–2021. Green iguana abundance declined from 103,020 (SE = 42,925) in August 2019 to 25,259 (SE = 9,485) in August 2020. However, abundance increased to 87,751 (SE = 34,706) in August 2021. Harvest rates declined from 0.629 between October 2018 and July 2019 to 0.568 in August 2019, 0.374 in August 2020, and 0.082 in August 2021. Green iguana abundance increased between 2020 and 2021 as harvesting declined due to diminishing returns to hunters paid a per green iguana harvested (Fig. 2). In addition to diminishing returns, hunters did not report harvested green iguanas in April 2020 due to a government-mandated coronavirus pandemic 40-day lockdown (Fig. 2). As expected from previous modeling simulations with harvest rates < 0.600 (see Rivera-Milán and Haakonsson 2020: Fig. 2a), the population recovered rapidly and surpassed 50,000 green iguanas between the August 2020 and 2021 surveys (Table 1).

Based on modeling simulations with *h* ~ Uniform (0.001, 0.900) to account for the possibility of highly variable harvest rates during 2022–2030, the mean posterior estimate of parameter *K* was 1,378,259 (SD = 298,802, median = 1,338,280, 2.5–97.5th percentiles = 927,055–1,953,958), the mean posterior estimate of parameter *r*_*max*_ was 1.323 (SD = 0.415, median = 1.354, 2.5–97.5th percentiles = 0.548–1.965), and the mean posterior estimate of parameter *h*_*msy*_ was 0.661 (SD = 0.207, median = 0.677, 2.5–97.5th percentiles = 0.274–0.983). Despite additional abundance estimates from the August 2020 and 2021 surveys, the posterior estimates of parameters *K*, *r*_*max*_ and *h*_*msy*_ were highly variable but similar to those reported by Rivera-Milán and Haakonsson (2020, Table 3). Model-based abundance predictions were also highly variable but similar to those previously reported (see Rivera-Milán and Haakonsson 2020: Fig. 2a). Therefore, based on modeling simulations, we need to keep harvest rates > 0.600 for desired abundance ≤ 50,000 green iguanas (Rivera-Milán and Haakonsson 2020), and we need to keep harvest rates > 0.700 for desired abundance ≤ 10,000 green iguanas (Table 2, Fig. 1a).

**Table 2:** Green iguana posterior mean and median estimates with 2.5th and 97.5th percentiles of predicted abundance for August 2030, based on the Bayesian state-space logistic model simulations with harvest rates between 0.001 and 0.900 during 2022–2030

Harvest rate^a^	Mean	Median	SD^b^	2.5th	97.5th
0.001–0.100	1,141,514	1,239,781	647,951	13	2,252,470
0.101–0.300	1,053,520	1,149,000	563,995	8	2,074,846
0.301–0.500	766,491	806,817	583,198	4	1,782,551
0.501–0.700	200,382	73,179	343,603	2	1,196,276
0.701–0.900	1470	18	19,441	0	8442
0.001–0.900	190,960	42,616	487,463	519	1,014,421

^a^*h* ~ Uniform (lower value, upper value)

^b^Markov Chain Monte Carlo standard deviations

More specifically, based on modeling simulations with *h* ~ Uniform (0.701, 0.900), we predict a green iguana abundance decline to 12,448 (SD = 54,571, median = 3027, 2.5–97.5th percentiles = 102–77,761) in August 2023 and 1470 (SD = 19,441, median = 18, 2.5–97.5th percentiles = 0–8442) in August 2030 (Table 2, Fig. 1a). In contrast, without harvesting (Fig. 1b), we predict a green iguana abundance increase to 417,026 (SD = 153,639, median = 392,866, 2.5–97.5th percentiles = 196,216–787,324) in August 2023 and 1,702,805 (SD = 302,447, median = 1,676,052, 2.5–97.5th percentiles = 1,676,052–2,390,200) in August 2029. Moreover, an unharvested green iguana population would likely reach and fluctuate around carrying capacity levels between August 2026 and 2029, lowering abundance predictability in August 2030 (i.e., mean = 1,387,508, SD = 1,614,523, median = 951,513, 2.5–97.5th percentiles = 1–5,480,424; Fig. 1b).

## Management implications and recommendations

Based on *r*_*max*_ 2.5–97.5th percentiles, the green iguana population has a doubling time of 4–15 months (i.e., *T* = ln[2]/*r*_*max*_ × 12). Therefore, with desired abundance ≤ 10,000 and estimated abundance averaging 87,751 in August 2021 (Table 1), we recommend keeping harvest rates > 0.700, which would imply harvesting > 61,426 green iguanas before the August 2022 survey. In addition, to assess population response to harvesting above *h*_*msy*_ (i.e., *h* > *r*_*max*_/2), we recommend monitoring twice per year, with a pre-reproduction survey in February and a post-reproduction survey in August 2023–2030. Long-term population monitoring and modeling are essential to guide decision-making and adapt the harvest strategy based on estimated and predicted abundance.

## Data Availability

Not applicable.
